# TIM3 expression on TILs is associated with poor response to neoadjuvant chemotherapy in patients with locally advanced triple-negative breast cancer

**DOI:** 10.1186/s12885-021-08054-6

**Published:** 2021-04-06

**Authors:** Neslihan Cabioglu, Semen Onder, Gizem Oner, Hüseyin Karatay, Mustafa Tukenmez, Mahmut Muslumanoglu, Abdullah İgci, Yeşim Eralp, Adnan Aydiner, Pınar Saip, Ekrem Yavuz, Vahit Ozmen

**Affiliations:** 1 Department of General Surgery, Istanbul Medical Faculty, Breast Surgery Service, Istanbul University, Istanbul, Turkey; 2 Department of Pathology, Istanbul Medical Faculty, Istanbul University, Istanbul, Turkey; 3 Current Address: Multidisciplinary Oncologic Centre Antwerp (MOCA), Antwerp University Hospital, Edegem, Belgium; 4 Current Address: Center for Oncological Research (CORE), University of Antwerp, Wilrijk, Belgium; 5 Department of Medical Oncology, Institute of Oncology, Istanbul University, Istanbul, Turkey; 6 Acibadem Maslak Hospital, Acibadem Health Group, Istanbul, Turkey

**Keywords:** Triple negative breast cancer, LAG-3, PD1, PDL1, CTLA4, T-cell immunoglobulin, Mucin domain-containing molecule 3 (TIM3), Tumor infiltrating lymphocytes (TILs)

## Abstract

**Background:**

The expression of immune checkpoint receptors (ICRs) on tumor-infiltrating lymphocytes (TILs) is associated with better response to immunotherapies via immune checkpoint inhibitors. Therefore, we investigated various ICR expressions on TILs in patients with locally advanced triple-negative breast cancer (TNBC) after neoadjuvant chemotherapy (NAC).

**Methods:**

Expressions of ICRs were examined immunohistochemically in surgical specimens (*n* = 61) using monoclonal antibodies for PDL-1, PD-1, TIM-3, LAG-3, and CTLA-4. Positivity was defined as staining > 1% on TILs.

**Results:**

The median age was 49 (24–76) years. The majority of patients were clinically T3–4 (*n* = 31, 50.8%) and clinically N1–3 (*n* = 58, 95.1%) before NAC. Of those, 82% were found to have CTLA-4 positivity, whereas PD1, PDL-1, LAG3, and TIM-3 expressions on TILs were 62.3, 50.9, 26.2, and 68.9%. A high expression of CTLA-4 was found to be associated with a better chemotherapy response (OR = 7.94, 95% CI: 0.9–70.12, *p* = 0.06), whereas TIM-3 positivity was contrarily associated with a worse chemotherapy response (OR = 0.253, 95% CI: 0.066–0.974, *p* = 0.047) as measured by the MDACC Residual Cancer Burden Index. At a 47-month follow-up, ypN0 (DFS; HR = 0.31, 95% CI: 0.12–0.83, *p* = 0.02 and DSS; HR = 0.21, 95% CI: 0.07–0.62, *p* = 0.005) and CTLA-4 high expression on TILs (DFS; HR = 0.38, 95% CI: 0.17–0.85, *p* = 0.019 and DSS; HR = 0.34, 95% CI: 0.15–0.78, p = 0.01) were found to be associated with improved survival.

**Conclusions:**

These findings demonstrate that CTLA-4, PD-1, PDL-1, and TIM-3 were highly expressed in TNBC. Based on these high expression patterns, further studies directed towards combined therapies are warranted in advanced TNBC in future.

## Background

As the interaction between the immune system and tumor progression is better understood, new treatment strategies have been developed [1, 2]. A better understanding of this relationship has changed the treatment strategy for many cancers with the development of checkpoint inhibitors [3, 4]. Many recent studies have demonstrated that the expression of immune checkpoint receptors (ICRs) on tumor-infiltrating lymphocytes (TILs) is associated with better response to immunotherapies, including immune checkpoint inhibitors [4–8].

Cytotoxic T Lymphocyte antigen 4 (CTLA-4), Programmed Cell Death 1 (PD-1), and Programmed Cell Death Ligand 1 (PD-L1, also known as B7-H1 or CD274) pathways are the best known immune checkpoint pathways for immune responses within the tumor microenvironment. Although considerable research has been devoted to CTLA-4, PD-1, and PD-L1, less attention has been paid to T cell immunoglobulin and mucin domain-containing protein 3 (TIM-3) and Lymphocyte-activation gene 3 (LAG-3) [9]. The CTLA-4 antibody ipilimumab was the first checkpoint inhibitor for use in advanced melanoma [7]. Subsequently, the Food and Drug Administration (FDA) approved inhibitors of PD-1 and/or PD-L1 in different types of cancer. On the other hand, research on TIM-3- and LAG-3- based treatment options or combined checkpoint inhibitor therapy is still ongoing [10–14].

Triple-negative breast cancer (TNBC) constitutes about 15–20% of all diagnosed breast cancers [15–17]. Although TNBC is typically characterized by a high degree of aggressiveness and poor prognosis, there are no approved molecularly targeted therapies for it [18, 19]. A recent meta-analysis reported an improved outcome in early TNBC with pathologic complete response (pCR) receiving neoadjuvant chemotherapy (NAC) [20]. Sequential administration of anthracyclines followed by taxanes has been the most common neoadjuvant approach in TNBC, and addition of carboplatin has also been considered to increase the pCR especially in those with BRCA mutations [21]. On the other hand, recent literature stresses that TNBC is more immunogenic than other breast cancer types [22–24]. Expression of ICRs on TILs has been associated with a better response to immunotherapies including immune checkpoint inhibitors (ICIs) as shown in advanced metastatic breast cancer by using combined modalities with chemotherapy [25]. Based on these encouraging results, recent studies focused on intensive investigations in neoadjuvant setting in combinations of ICIs with chemotherapeutic agents including anthracyclines, taxanes and platinum [26].

While early-phase studies of the immune checkpoint inhibitors in TNBC have shown evidence of activity, it is still a matter of debate as to which immune checkpoint marker will be used in treatment or which immune checkpoint marker has a prognostic value. Therefore, we investigated the expression of various new emerging ICRs, including TIM-3 and LAG-3, along with the best known PDL-1, PD-1, and CTLA-4 in patients with TNBC receiving NAC to explore their predictive and prognostic significance.

## Methods

The study included 61 patients with triple-negative locally advanced breast cancer (LABC) who underwent surgery following NAC at the Istanbul University, Faculty of Medicine, Department of General Surgery, from July 2002 to January 2018. The study was approved by the ethical committee of Istanbul University, Istanbul Medical Faculty.

### Patients and therapy

Patients with residual disease and pCR, who had received NAC due to the locally advanced TNBC were included in the study. The majority of patients (*n* = 57) received anthracycline and taxane containing NAC protocols, whereas 4 (6.6%) had an anthracycline-based regimen and 5 (6.6%) received additional carboplatine or cisplatine with/without gemcitabine or capecitabine. Of those, 45 (73.8%) underwent mastectomy and 26 (26.2%) had breast-conserving surgery. Furthermore, 53 patients (86.9%) underwent axillary dissection with/without sentinel lymph node biopsy (SLNB), whereas only 8 patients (13.1%) had SLN alone following completion of NAC. All patients received adjuvant radiotherapy to the chest wall for mastectomy and the whole breast for breast-conserving surgery with axillary (level I-II-III) and supraclavicular lymph node region irradiation following surgery.

All patients with TNBC had estrogen receptor and progesterone receptor positivity < 1%, and HER2 negativity was determined according to the ASCO/CAP guidelines. The absence of a residual invasive tumor in the resected breast specimen and in the regional lymph nodes after completion of NAC was defined as pCR. The chemotherapy response was evaluated using the “MD Anderson Cancer Center (MDACC) Residual Cancer Burden Index” (www3.mdanderson.org/app/medcalc/index.cfm.pagename = jsconvert) [27]. The residual cancer burden index was calculated from the parameters in pathology reports, including primary tumor bed area size, overall cancer cellularity, and percentage of in situ cancer. The MDACC Residual Cancer Burden Index classification implies a worsening chemotherapy response from “Class 0” to “Class 3”. “Class 0” was considered a pathological complete response, whereas “Class 3” was considered chemotherapy-resistant. Briefly, response to chemotherapy is considered good if Class 0 or 1 and not as good if Class 2 or 3. Clinicopathologic data included age, tumor characteristics, operation type, clinical and pathological stage, histologic grade, and lymph node status, and any local and/or systemic recurrences and follow-up time were analysed.

### Immunohistochemical analysis of immune checkpoint receptors

Expression of ICRs was studied in paraffin blocks of surgical specimen following NAC, respectively. The most suitable tumor block that included tumor-infiltrating lymphocytes (TILs) was selected for immunostaining. Immunohistochemical evaluation was performed with an automatic Ventana BenchMark (Ventana Medical Systems, Tucson, AZ, USA) slide staining device. CTLA-4, LAG-3, PD-L1, PD-1, and TIM-3 expression was assessed on 5-μm formalin-fixed paraffin-embedded slides on tumor and TILs. Sections were incubated with primary antibodies for PDL-1 (rabbit mAb; SP263 clone kit, Ventana Medical Systems, Tucson, AZ, USA) at 1:100 dilution, for PD-1 (rabbit mAb; AC0255RUO, Epitomics, Abcam, UK) at 1:200 dilution, for CTLA-4 (rabbit policlonal Ab; PA5–23967, Thermo Fisher Scientific, UK) at 1:100 dilution, for LAG-3 (rabbit policlonal Ab D2G40™; Cell Signaling Tech., MA, USA) at 1:200 dilution, and for TIM-3 (XP rabbit, D5D5R™, Cell Signaling Tech., MA, USA) at 1:200 dilution. Tonsil tissue was used as a control in the immunohistochemistry procedure for all of these antibodies.

The intensity and staining percentage were noted for each immune checkpoint receptor showing membranous staining. The intensity was scored as weak, moderate, or strong. Different staining percentages from 1 to 20% depending on the median values for each marker with or without staining intensity were tested for any significant associations with prognosis. CTLA-4 and PD-L1 positivity were defined as membranous staining > 1% on either tumor or TILs, whereas LAG-3, TIM-3, and PD-1 positivity on TILs was defined as membranous staining > 1% on TILs. All micrographs were taken by using an integrated digital camera (Olympus DP71, Japan) on a light microscope (Olympus BX51, Japan).

### Statistical analysis

The statistical analyses were performed using the statistical software program SPSS 25 (Statistical Package for Social Sciences; SPSS, IBM Corp., Armonk, NY, USA). A *p*-value equal to or less than 0.05 was considered statistically significant. Categorical variables were evaluated by Chi Fisher’s exact tests including Pearson chi-square, continuity correction, and Fisher’s exact tests in two-tailed univariate analyses. Binary logistic regression analysis was used to estimate the significant associations linked to chemotherapy response. Furthermore, the Pearson correlation test was used to assess the correlations of continuous variables.

Disease-free and disease-specific survival rates were calculated by Kaplan-Meier analyses, and factors associated with survival were tested by the log-rank test. Disease-free survival (DFS) was analysed by considering local and systemic metastases, and disease-specific survival (DSS) rates were analysed by considering breast-cancer-related mortality. A log-rank test was used to compare the prognostic effect of different variables. Variables that were found to be significant in univariate analyses were further evaluated in a Cox regression model to calculate the hazard ratio of factors associated with poor prognosis.

## Results

The patients’ median age was 49 (24–76) years. Of 61 patients, 31 (50.8%) were clinically T3–4 (55%), whereas the remainder were cT1–2 (49.2%). Almost all of them were clinically N1–3 (*n* = 58, 95.1%) presenting mostly with cN1 disease (*n* = 36, 59%). Twenty-three patients (37.7%) were found to have axillary pathologic complete response (ypN0), while 18 had (29.5%) ypN1, 11 (18%) had ypN2, and 9 (14.8%) had ypN3 after NAC. Among patients having a residual tumor (*n* = 51), the vast majority had invasive ductal carcinoma (*n* = 41, 80.4%), while 3 (5.9%) had invasive lobular carcinoma, 1 (1.9%) had a mix of invasive ductal and lobular, and 6 (11.8%) had metaplastic carcinoma.

Response to chemotherapy and histopathological analysis was evaluated in 60 patients by the “MDACC Residual Cancer Burden Index”. One patient with a residual tumor in the lymphovascular area was excluded from this analysis. The median score was 2.72 (0–5.1). According to the MDACC Residual Cancer Burden classification, 26 patients (43.3%) had Class III as non-responders, 21 patients (35%) had Class II as a partial response, 4 (6.7%) had Class I as a near-complete response, and 9 (15%) had Class 0 with a pathologic complete response.

### Immunohistochemical expression of immune checkpoint receptors

The median values of staining percentages for PD-L1, PD-1, CTLA-4, LAG-3, and TIM-3 in TILs were 1 (0–50), 2 (0–70), 7.5 (0–60), 0 (0–60), and 2 (0–50), respectively, whereas the staining percentages of tumoral expressions of PD-L1 and CTLA-4 were 0.5 (0–30) and 7.5 (0–70), respectively.

Significant correlations were detected between different immune checkpoint receptors on TILs in the Spearman correlation analysis (Table 1). Expression levels of PD-L1 on TILs were significantly correlated with PD-L1 expression on the tumor (p = < 0.001), and PD-1 (*p* = 0.007), LAG-3 (*p* = 0.005), whereas PD-L1 expression on TILs was found to be correlated with TIM-3 expression levels on TILs (*p* = 0.055) that did not reach the statistical significance (Table 1). Expression of PD-1 has also shown significant correlations with CTLA-4 (*p* < 0.001) and TIM-3 on TILs (*p* = 0.001), whereas CTLA-4 expression on TILs correlated with CTLA-4 expression on the tumor (p < 0.001) and TIM-3 expression on TILs (*p* = 0.018). Finally, LAG-3 expression on TILs was found to be significantly correlated with TIM-3 expression on TILs (*p* = 0.033).

**Table 1:** Spearman correlations (SC) of the immune checkpoint receptor (=ICR) expressions in TILs (*n* = 61)

		PDL-1	PDL-1_**tumor**_	PD1	LAG3	TIM3	CTLA-4_**TILs**_	CTLA-4_**TM**_
**PDL-1**_**tumor**_	**r**	0.772						
	***P-value***	< 0.001**						
	**n**	48						
**PD1**	**r**	0.354	0.352					
	***P-value***	0.007**	0.014*					
	**n**	57	48					
**LAG3**	**r**	0.370	0.476	0.174				
	***P-value***	0.005**	0.001**	0.179				
	**n**	57	48	61				
**TIM3**	**r**	0.256	0.236	0.407	0.274			
	***P-value***	0.055	0.106	0.001**	0.033*			
	**n**	57	48	61	61			
**CTLA-4**_**TILs**_	**r**	0.148	0.068	0.460	0.037	0.301		
	***P-value***	0.272	0.648	< 0.001**	0.776	0.018*		
	**n**	57	48	61	61	61		
**CTLA-4**_**TM**_	**r**	0.131	0.106	0.248	0.251	0.277	0.486	
	***P-value***	0.374	0.472	0.076	0.073	0.047*	< 0.001**	
	**n**	48	48	52	52	52	52	
**MDACC Score**	**r**	−0.061	−0.010	0.153	−0.061	0.062	0.008	0.119
	***P-value***	0.653	0.947	0.244	0.645	0.637	0.953	0.405
	**n**	56	47	60	60	60	60	51

*p < 0.05, **p < 0.01, r: Spearman Correlations

MDARB-Index: MD Anderson Residual Cancer Burden Index

**Fig. 1:**
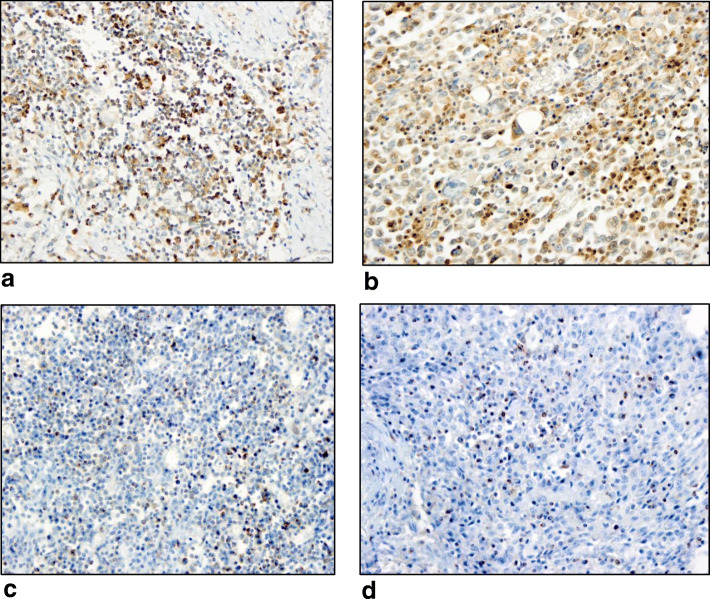
**a.** High PD1 immunohistochemical expression on TILs (× 400, HPF. **b.** High CTLA4 immunohistochemical expression on TILs and tumor (× 400, HPF). **c.** Immunohistochemical expression of LAG-3 on TILs (× 400, HPF). **d.** Immunohistochemical expression of TIM3 on TILs (× 400, HPF)

A cut-off > 1% staining percentage regardless of staining intensity was considered positive for each ICR. Of 61 cases, TIL-associated positivities for the ICRs were as follows: PD-L1 (50.9%), PD-1 (62.3%), LAG-3 (26.2%), TIM-3 (52%), and CTLA-4 (82%) (Table 2, Fig. 1). The higher levels of staining percentages based on the median values of each marker with/without staining intensity were tested for any significant associations with survival. High CTLA-4 expression was considered for any staining > 15%, and any medium and strong staining if < 15%.

**Table 2:** Associations of immune check point receptor expression in TILs with neoadjuvant chemotherapy response as calculated “MD Anderson Cancer Center (MDACC) Residual Cancer Burden Index” in TNBC patients

Expression in TILs (>%1)	N = 61 (%)	pCR(+) vs (−)^a^	***P***- value	Class 0/I vs II/III^a^	***P***- value	Class 0/I/II vs III^a^	***P***- value
PD-L1 (+) vs other	29/57 (50.9%)	5/9 (56%) vs 24/47 (51%)	0.999	6/11 (55%) vs 23/45 (51%)	0.999	19/31 (61%) vs 10/25 (40%)	0.188
PD1 (+) vs other	37/61 (62%)	5/9 (56%) vs 32/51 (63%)	0.721	7/13 (54%) vs 30/47 (64%)	0.739	19/34 (56%) vs 18/26 (69%)	0.432
LAG3 (+) vs other	16/61 (26%)	2/9 (22%) vs14/51 (28%)	0.999	3/13 (23%) vs 13/47 (28%)	0.999	11/34 (32%) vs 5/26 (19%)	0.378
PD1&LAG3 (+) vs other	14/61 (23%)	2/9 (22%) vs 12/51 (24%)	0.999	3/13 (23%) vs 11/47 (23%)	0.999	9/34 (26%) vs 5/26 (19%)	0.555
TIM3 (+) vs other	42/61 (69%)	2/9 (22%) vs 39/51 (77%)	**0.003**	6/13 (46%) vs 35/47 (75%)	0.108	25/34 (74%) vs 16/26 (62%)	0.478
PD1&TIM3 (+) vs other	31/61 (51%)	2/9 (22%) vs 28/51 (55%)	0.145	4/13 (31%) vs 26/47 (55%)	0.209	16/34 (47%) vs 14/26 (54%)	0.794
CTLA-4 (+) vs other	50/61 (82%)	9/9 (100%) vs 40/51 (78%)	0.189	12/13 (92%) vs 37/47 (79%)	0.427	30/34 (88%) vs 19/26 (73%)	0.182
CTLA-4^b^(+) vs other	43/61 (70.5%)	9/9 (100%) vs 33/51 (65%)	**0.047**	12/13 (92%) vs 30/47 (64%)	0.084	26/34 (77%) vs 16/26 (62%)	0.334
PD1&CTLA-4 (+) vs other	33/61 (54%)	5/9 (56%) vs 27/51 (53%)	0.999	7/13 (54%) vs 25/47 (53%)	0.999	18/34 (53%) vs 14/26 (54%)	0.999

Tumor infiltrating (stromal) lymphocytes (=TILs)

^a^ One patient with a residual tumor in the lymphovascular area has been excluded

^b^CTLA-high expression: > 15%&any moderate or strong staining if < 15%

Associations of ICR positivities and response to chemotherapy were tested by using the MDACC Residual Cancer Burden Index. A high expression of CTLA-4 (*p* = 0.047) and TIM-3-negativity (*p* = 0.003) were found to be significantly associated with pCR (Table 2). When the MDACC Residual Cancer Burden Index “Class 0&1” vs “Class 2&3” were compared, high expression of CTLA-4 on TILs was found to be associated with a better chemotherapy response (OR = 7.94, 95% CI: 0.9–70.12, *p* = 0.06), whereas TIM-3 positivity was contrarily associated with a worse chemotherapy response (OR = 0.253, 95% CI: 0.066–0.974, *p* = 0.047). No significant associations could be found between other ICR expressions and response to NAC.

### Outcome and survival analyses

**Fig. 2:**
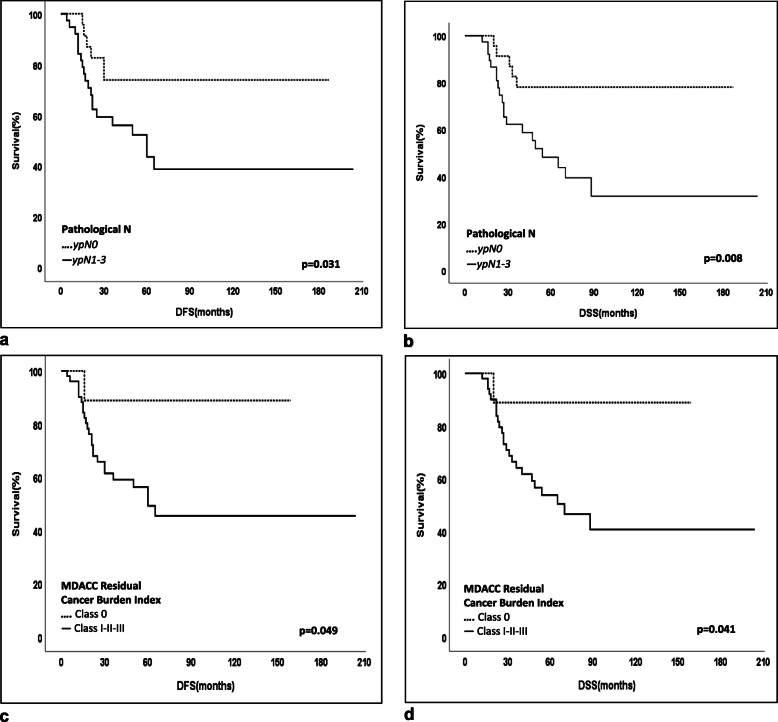
**a**. 5-year disease-free survival (DFS) in patients with ypN0, 73.9% vs ypN+, 43.6%; *p* = 0.031. **b.** 5-year disease-specific survival (DSS) in patients with ypN0, 78% vs ypN+, 48.1%; *p* = 0.008. **c.** 5-year disease-free survival (DFS) in patients with MDACC Residual Cancer Burden Index Class 0, 88.9% vs MDACC Residual Cancer Burden Index Class I-III, 49.3%; *p* = 0.049. **d.** 5-year disease-free survival (DSS) in patients with MDACC Residual Cancer Burden Index Class 0, 88.9% vs MDACC Residual Cancer Burden Index Class I-III, 53%; *p* = 0.041

**Fig. 3:**
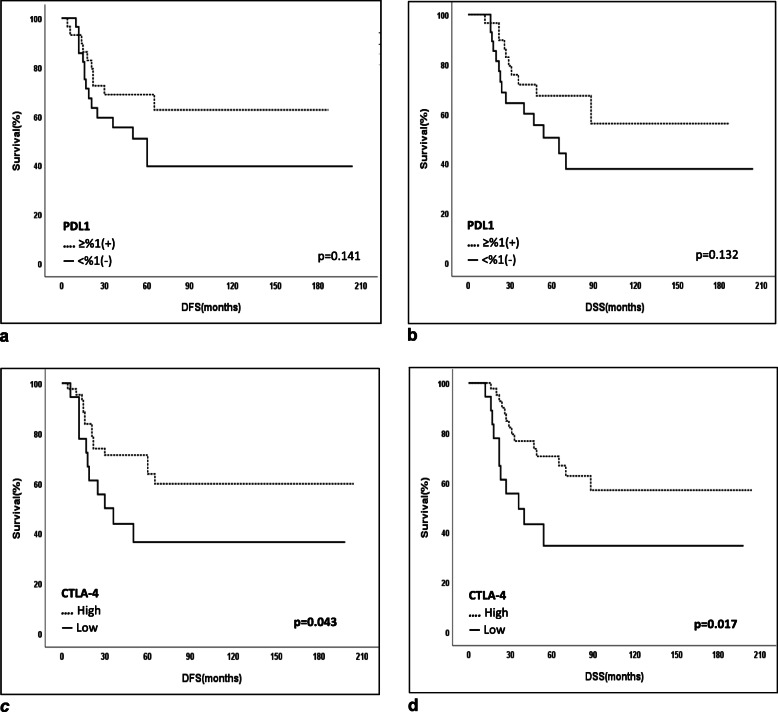
**a.** 5-year disease-free survival (DFS) in patients with PDL-1 (≥1%), 62.5%, vs PDL-1 (< 1%), 39.5%; *p* = 0.141. **b.** 5-year disease-specific survival (DSS) in patients with PDL-1 (≥1%), 67.2%, vs PDL-1 (< 1%), 50.4%; *p* = 0.132. **c.** 5-year disease-free survival (DFS) in patients with CTLA4-High*, 63.7%, vs CTLA4-Low, 36.5%; *p* = 0.043 (* > 15% & any moderate/strong staining if < 15%). **d.** 5-year disease-specific survival (DSS) in patients with CTLA4-High, 70.5%, vs CTLA4-Low, 43.2%; *p* = 0.017

**Fig. 4:**
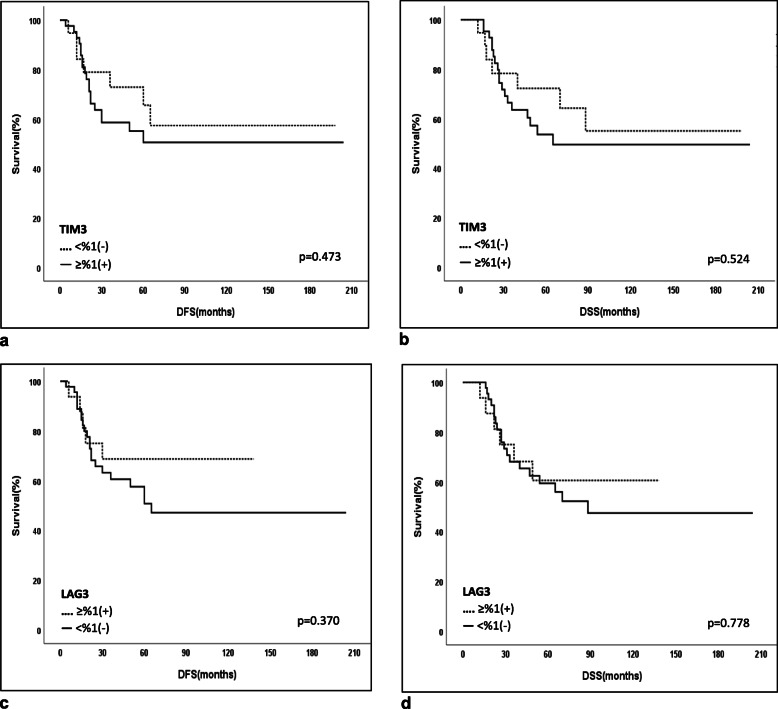
**a.** 5-year disease-free survival (DFS) in patients with TIM3 (≥1%), 50.5%, vs TIM3 (< 1%), 65.6%; *p* = 0.473. **b.** 5-year disease-specific survival (DSS) in patients with TIM3 (≥1%), 53.6%, vs TIM3 (< 1%), 72.3%; *p* = 0.524. **c.** 5-year disease-free survival (DFS) in patients with LAG3 (≥1%), 68.8% vs LAG3 (< 1%), 50.8%; *p* = 0.37. **d.** 5-year disease-specific survival (DSS) in patients with LAG3 (≥1%), 60.6%, vs LAG3 (< 1%), 59.4%; *p* = 0.778

At the median follow-up time of 47 months (12–204), the 5-year disease-free survival (DFS) and disease-specific survival (DSS) were 55 and 59.8% for the whole cohort, respectively. Associations between clinicodemographic and pathological characteristics and survival revealed that patients with ypN0 or a pCR or with a Class 0&I MDACC Residual Cancer Burden Index indicating a good response to NAC were found to have a better 5-year disease-free survival (DFS) and disease-specific survival (DSS) (Table 3, Fig. 2). Positivities of PD-L1, PD-1, LAG-3, TIM-3, and CTLA4 and different combinations of ICR coexpressions including PD-L1/LAG3, etc. and different cut-off levels based on staining percentage and/or staining intensity were tested for any significant associations with survival as shown in Table 4. High CTLA-4 expression on TILs was the only significant factor associated with an improved 5-year disease-free survival (DFS) and disease-specific survival (DSS) (DFS: CTLA4-low, 36.5% vs CTLA4-high, 63.7%; *p* = 0.043; DSS: CTLA4-low, 43.2% vs CTLA4-high, 73.5%; *p* = 0.017) (Fig. 3). A multivariate logistic regression analysis demonstrated that ypN0 (DFS; HR = 0.31, 95% CI: 0.12–0.83, *p* = 0.02 and DSS; HR = 0.21, 95% CI: 0.07–0.62, *p* = 0.005) and CTLA-4 high expression (DFS; HR = 0.38, 95% CI: 0.17–0.85, *p* = 0.019 and DSS; HR = 0.34, 95% CI: 0.15–0.78, p = 0.01) were found to be associated with a good prognosis (Table 5, Fig. 3). No significant difference could be found in DFS and DSS rates in regards to PD-L1, PD-1, TIM-3, and LAG-3 expressions on TILs (Table 4, Fig. 4). Furthermore, CTLA4 tumoral expression (> 1%) was seen in 25 of 52 patients with residual tumor after NAC (48%). No difference could be found between patients with CTLA4-positivity and CTLA4-negativity on tumor in 5-year DFS (CTLA-4(+), 51.4% vs CTLA-4(−), 53%, *p* = 0.846) and DSS rates (CTLA4(+), 51.1% vs CTLA4(−), 58.2%, *p* = 0.814).

**Table 3:** Survival analyses associated with clinicodemographic or pathological patient characteristics

Characteristics	n	5-Year DFS(%)	p	5 Year DSS(%)	p
Age ≤ 50	31	55.8	0.999	58.2	0.895
Age > 50	30	54.6		61.4	
**cT (Clinical = Physical exam&Radiological)**			0.624		0.526
T1–2	30	55.7		62.9	
T3–4	31	53.9		57.2	
**cN**			0.719		0.441
N0–1	39	55.6		64.0	
N2–3	22	52.3		52.1	
ypN*			0.031*		0.008*
ypN0	23	73.9		78.0	
ypN1–3	38	43.6		48.1	
**Pathological findings after NAC**			0.367		0.230
Invasive ductal carcinoma	40	48.0		54.6	
Other	21	66.0		69.7	
*MDACC Residual Cancer Burden Index			0.049*		0.041*
Class 0 (=pathologic complete response)	9	88.9		88.9	
Class I-II-III	51	49.3		53.0	
*MDACC Residual Cancer Burden Index			0.038*		0.037*
Class 0–1 (=pathologic complete response)	13	83.9		83.9	
Class II-III	47	48.1		52.7	

*NAC* Neoadjuvant chemotherapy, *p < 0.05

**Table 4:** Survival analyses associated with immune check point receptor expression in patients with TNBC after NAC

Expressions in TILs	N	5-year Disease Free Survival (DFS) (%)	p	5-year Disease Specific Survival (DSS) (%)	p
**PDL-1** ≥ 1% (+) vs < 1% (−)	29/28	62.5% vs 39.5%	0.141	67.2% vs 50.4%	0.132
**PDL-1** ≥ 5% (+) vs < 5% (−)	25/32	65.3% vs 38.9%	0.096	66.2% vs 53.3%	0.213
**PDL-1 High (**≥10%**) vs Low (**< 10%**)**^a^	26/31	66.2% vs 37.5%	0.062	67.6% vs 51.5%	0.145
**PDL-1** ≥ 10% (+) vs < 10% (−)	18/39	67.4% vs 42.9%	0.087	69.9% vs 54.0%	0.197
**PDL-1** ≥ 20% (+) vs < 20% (−)	15/42	50.0% vs 51.3%	0.996	51.9% vs 62.1%	0.651
**PD-1** ≥ 1% (+) vs < 1% (−)	38/23	50.6% vs 61.6%	0.121	51.4% vs 62.7%	0.082
**PD-1** ≥ 5% (+) vs < 5% (−)	26/35	54.2% vs 56.1%	0.593	58.1% vs 69.1%	0.270
**PD-1 High(**≥10%**) vs Low(**< 10%**)**^a^	24/37	56.8% vs 54.8%	0.517	59.6% vs 66.9%	0.263
**PD-1** ≥ 10% (+) vs < 10% (−)	10/51	67.5% vs 49.4%	0.193	72.9% vs 57.4%	0.439
**PD-1** ≥ 20% (+) vs < 20% (−)	6/55	83.3% vs 52.2%	0.277	83.3% vs 59.5%	0.570
**LAG-3** ≥ 1% (+) vs < 1% (−)	16/45	68.8% vs 50.8%	0.370	60.6% vs 59.4%	0.778
**LAG-3** ≥ 5% (+) vs < 5% (−)	7/54	57.1% vs 55.3%	0.804	71.4% vs 62.9%	0.339
**LAG-3 High(**≥10%**) vs Low(**< 10%**)**^a^	6/55	66.7% vs 53.9%	0.815	66.7% vs 61.3%	0.683
**TIM3** ≥ 1% (+) vs < 1% (−)	42/19	50.5% vs 65.6%	0.473	53.6% vs 72.3%	0.524
**TIM3** ≥ 5% (+) vs < 5% (−)	15/46	52.8% vs 55.4%	0.947	62.7% vs 59.2%	0.959
**TIM3 High (**≥10%**) vs Low (**< 10%**)**^a^	14/47	57.1% vs 55.2%	0.314	68.8% vs 60.5%	0.535
**CTLA-4** ≥ 1% (+) vs < 1% (−)	50/11	57.2% vs 45.5%	0.272	63.2% vs 45.5%	0.209
**CTLA-4** ≥ 5% (+) vs < 5% (−)	48/13	55.9% vs 53.8%	0.595	61.8% vs 53.8%	0.481
**CTLA-4 High(**≥15%**) vs Low(**< 15%**)**^b^	43/18	63.7% vs 36.5%	**0.043**	70.5% vs 43.2%	**0.017**
**CTLA-4** ≥ 10% (+) vs < 10% (−)	29/32	60.1% vs 51.1%	0.429	67.4% vs 53.6%	0.468
**CTLA-4** ≥ 15% (+) vs < 15% (−)	19/42	74.9% vs 46.6%	0.103	74.0% vs 53.8%	0.245
**CTLA-4** ≥ 20% (+) vs < 20% (−)	18/43	72.9% vs 48.1%	0.165	72.1% vs 55.0%	0.373
**PD1/PDL-1** ≥ 1% (+) vs < 1% (−)	22/38	52.5% vs 51.3%	0.908	58.4% vs 60.4%	0.997
**PD1/LAG3** ≥ 1% (+) vs < 1% (−)	14/47	64.3% vs 52.4%	0.619	64.0% vs 61.1%	0.903
**PD1/TIM3** ≥ 1% (+) vs < 1% (−)	31/30	48.5% vs 62.2%	0.273	49.4% vs 75.2%	0.204
**PD1/CTLA4** ≥ 1% (+) vs < 1% (−)	33/28	58.6% vs 50.2%	0.966	59.9% vs 65.0%	0.984

^a^ > %10&any moderate/strong staining vs other

^b^ > %15&any moderate/strong staining vs other

bold: *p* < 0.05

**Table 5:** Multivariate Cox Regression Analysis

	Disease Free Survival		Disease Specific Survival	
**Factors**	**HR (95%CI)**	***P*** **value**	**HR (95%CI)**	***P*** **value**
**ypN**		**0.02**		**0.005**
N0	0.31 (0.12–0.83)		0.21 (0.07–0.62)	
N1–3	Reference [1]		Reference [1]	
**CTLA-4**		**0.019**		**0.01**
Low	Reference [1]		Reference [1]	
High^a^	0.38 (0.17–0.85)		0.34 (0.15–0.78)	

**Cox Regression** (Step-2, Method = Forward Stepwise)

Variables not in the Equation: PDL-1(<%1 vs ≥ %1), MDACC Residual Cancer Burden Index (Class 0 vs Class I-III)

Hazard ratio (HR) are presented with their 95% confidence interval (CI) and the p-value

^a^ > 15% & any moderate/strong staining if < 15%

## Discussion

Targeting immune checkpoints in cancer immunotherapy has made substantial progress in the treatment of certain solid cancers, including breast cancer, focusing on TNBC as the most immunogenic and aggressive subtype. However, long-term durable responses were obtained in only 10 to 30% of patients, while some patients develop resistance to the current immunotherapy regimens and may benefit from further combinatorial blockade targeting potential new immune checkpoints, such as LAG-3 or TIM-3 [28]. Furthermore, a significant proportion of patients suffer from immune-related adverse effects when treated with CTLA-4, PD-1, or PD-L1 inhibitors. The hypothesis regarding whether combining the currently known inhibitors targeting PD-1, PD-L1, or CTLA-4 with the promising new inhibitors against LAG-3 and TIM-3 may enhance therapeutic efficacy while reducing the side effects is currently under investigation in clinical trials [14]. In this study, the predictive and prognostic significance of various ICRs including PD-L1, PD-1, and CTLA-4, along with the new emerging receptors TIM3 and LAG3, was tested in locally advanced TNBC. We briefly report here that CTLA-4 was the only biomarker as ICR associated with a better chemotherapy response and better outcome, whereas TIM-3 expression was negatively correlated with response to NAC.

Multiple ICRs, including PD-1, CTLA-4, TIM-3, and LAG-3, were found to be upregulated in breast cancer tissues as compared to normal breast tissue [29]. Despite some inconsistent results in the literature, the majority of the studies indicated an improved prognosis and better response to immunotherapy in patients with TNBC having PD-1 or PD-L1 expression on TILs [30, 31]. In a recent meta-analysis including 47 studies with a total of 14,367 patients who had breast cancer, Huang et al. [31] reported that PD-L1+ expression on TILs was associated with a better DFS (HR = 0.45, 95% CI: 0.28–0.73, *p =* 0.001) and OS (HR = 0.41, 95% CI: 0.27–0.63, *p* < 0.0001). Contrarily, PD-L1 expression on the tumor was found to be associated with shorter DFS (HR = 1.43, 95% CI: 1.21–1.70, p < 0.0001) and overall survival (OS, HR = 1.58, 95% CI: 1.14–2.20, *p* = 0.006). In our study, we could not find any significant difference in DFS or DSS between patients with or without PD-L1 or PD-1 expression on TILs. Though there was a trend towards a better prognosis in patients with PD-L1 expression on TILs, it did not reach statistical significance. Furthermore, we found a significant correlation between tumoral PD-L1 expression and PD-1 expression on TILs similar to the findings of the meta-analysis of Huang et al. [31].

The first inhibitory immune checkpoint receptor studied in the clinic, CTLA-4, was previously shown to be present as a surface molecule on solid tumor cell lines including breast carcinomas and osteosarcomas [32]. Though the exact function of expression of CTLA-4 on tumor cells is unknown, incubating the cells with CTLA-4 ligands CD80 or CD86 induced apoptosis in human osteosarcoma cell line HOS cells through caspase-3 and caspase-8 activation [32]. Yu et al. showed that CTLA-4^+^ lymphocyte density high was an independent predictor of better DFS (HR = 0.315, 95% CI: 0.15–0.658, *p* = 0.002) and OS (HR = 0.313, 95% CI: 0.139–0.703, *p* = 0.005), whereas tumor CTLA-4^high^ was an independent predictor of shorter DFS (HR = 2.176, 95% CI: 1.084–4.437, *p* = 0.029) and OS (HR = 2.820, 95% CI: 1.337–5.95, *p* = 0.007) in 130 patients with operable breast cancer [33]. In our study, almost half of the tested tumors expressed tumoral CTLA-4 that significantly correlated with CTLA-4 and TIM3 expression on TILs. In concordance with the study of Yu et al., we demonstrated that CTLA-4 high expression on TILs (DFS; HR = 0.38, 95% CI: 0.17–0.85, *p* = 0.019 and DSS; HR = 0.34, 95% CI: 0.15–0.78, p = 0.01) and ypN0 (DFS; HR = 0.31, 95% CI: 0.12–0.83, p = 0.02 and DSS; HR = 0.21, 95% CI: 0.07–0.62, p = 0.005) were independently found to be associated with improved survival in multivariate analysis. However, we could not find any prognostic significance of tumoral CTLA-4 expression in our study consisting of patients with locally advanced TNBC, whereas Yu’s study included operable patients with both luminal and non-luminal tumors. Furthermore, high expression of CTLA-4 on TILs was significantly associated with a pCR and a better chemotherapy response (OR = 7.94, 95% CI: 0.9–70.12, *p* = 0.06) based on the MDACC Residual Cancer Burden Index in our cohort with TNBC. Similarly, Kaewkangsadan et al. found a significant association between the presence of abundant levels of TILs, and CD4^+^, CD8^+^, CTLA-4^+^ stromal T cells, and pCR in 33 patients with locally advanced breast cancer undergoing NAC, suggesting that CTLA-4^+^ TILs may play a role in creating a good response to chemotherapy [34].

However, TIM-3, which has been studied as a newly emerging and promising coinhibitory molecule expressed on TILs, was contrarily found to be associated with a worse chemotherapy response (OR = 0.253, 95% CI: 0.066–0.974, *p* = 0.047) as measured by the MDACC Residual Cancer Burden Index in our study. Using an anti-TIM-3 antibody increased the response to paclitaxel chemotherapy in MMTV-PyMT transgenic mice as studied in models of triple-negative and luminal B disease, with no evidence of toxicity [35]. Yashinka et al. further suggested that the TIM-3-galectin-9 pathway may be involved in the immune escape of cancer cells [36]. They reported higher expression of both galectin-9 and TIM-3 in breast cancer tissues compared to healthy breast tissues of the same patients by demonstrating the colocalization of these proteins in breast tumors. Furthermore, Burugu et al. recently investigated the immunohistochemical expression of TIM-3 in 3992 breast cancer specimens in tissue microarray along with other biomarkers, including CD8, PD-1, PD-L1, and LAG-3 [37]. In multivariate analysis, the expression of TIM-3, PD-1, or LAG-3 on TILs was found to be an independent favorable prognostic factor among ER-negative patients. Similarly, Byun et al. reported a better prognosis in patients with TIM-3 expression on TILs in TNBC [38]. These studies also demonstrated that the presence of TIM-3 on TILs was significantly associated with the expression of other ICRs, including PD-1 and LAG-3 on TILs and PD-L1 on tumors. Our findings have shown that the majority of patients (69%) had TIM-3 positivity, and similarly indicated significant correlations between TIM-3 expression on TILs and LAG-3 or PD-1 or PD-L1 on TILs. Even though TIM-3 expression was negatively correlated with response to NAC in our study, we could not demonstrate any prognostic significance of these ICRs in our cohort of patients with TNBC following NAC presenting with more advanced disease compared to other studies. The prognostic significance of TIM-3 expression should be therefore studied in cases with TNBC including different stages in future.

Another novel coinhibitory receptor, LAG-3, which negatively regulates T-cell activation, has been reported to be present at a lower expression rate (18%) in early-stage TNBC as compared to PD-1 expression on TILs (30%), whereas coexpression of both receptors was found in 15.4% of breast-tumor-associated TILs [39]. Similarly, expressions of PD-1 and LAG-3 and concurrent expressions of PD-1 and LAG-3 on TILs were shown to be 62, 26, and 23%, respectively, in our study. However, the biological significance of the concurrent expression of inhibitory receptors such as PD-1 and LAG-3 is unknown. No prognostic significance could be found in patients with LAG-3 expression alone or concurrent LAG-3 and PD-1 expression in the study of Bottai et al. in concordance with our study. However, a recent meta-analysis of 15 studies including different cancers indicated that LAG-3 expression was associated with better OS (HR = 0.81, 95% CI: 0.66–0.99, *p* = 0.04), with a greater magnitude in early-stage malignancies (HR = 0.73, 95% CI: 0.60–0.88) and an improved DFS in breast cancer in a subgroup analysis (HR = 0.64, 95% CI: 0.42–0.98) [40]. Finally, Burugu et al. [41] studied LAG-3 expression in 4322 breast cancer specimens by immunohistochemistry and reported an improved outcome in patients with LAG-3 expression on TILs with a breast-cancer-specific survival (HR = 0.71, 95% CI: 0.56–0.90), particularly among estrogen receptor-negative patients (HR = 0.50, 95% CI: 0.36–0.69). Furthermore, concurrent LAG-3 expression with PDL1 and PD1 was 53 and 61%, respectively. These values seem to be higher than our findings and Bottai’s study [39], which might be due to the different clinicopathological characteristics and stages of patients in the study cohorts.

We recently have studied the expressions of immune checkpoints receptors including PD-1, LAG-3, TIM-3, TIGIT and CTLA-4 on CD8 T lymphocytes and Natural Killer (NK) cell subsets obtained from TILs of patients with LABC following NAC by flow-cytometry [42]. Our results suggested that HER2+ patients were more likely to have increased TIM-3 expressions on cytotoxic CD8- T cells, and patients with a younger age and advanced tumor burden are more likely to express ICRs on TILs compared to others. More studies are warranted to understand the precise role of LAG-3 or TIM-3 coexpressions on TILs by using different techniques including flow-cytometry or multiplex immunohistochemistry stainings in larger cohorts of TNBC with different stages.

## Conclusion

Our findings indicate that novel immunological biomarkers, including TIM-3 and LAG-3, were highly expressed in TNBC after NAC similar to CTLA-4, PD-1, and PD-L1 showing different predictive and prognostic features. Based on these high expression patterns, combined immune checkpoint inhibitor therapies via CTLA-4 and/or PD-1 or PDL-1 along with TIM-3 or LAG-3 are to be investigated in future studies, which may improve the prognosis and pathologic complete response rates of patients undergoing chemotherapy [6, 9–12, 14, 43, 44].

## Data Availability

The datasets during and/or analysed during the current study available from the corresponding author on reasonable request.

## Abbreviations

ICR: Immune checkpoint receptors
TILs: Tumor-infiltrating lymphocytes
TNBC: Triple-negative breast cancer
NAC: Neoadjuvant chemotherapy
CTLA-4: Cytotoxic T Lymphocyte antigen 4
PD-1: Programmed Cell Death 1
PD-L1: Programmed Cell Death Ligand 1
TIM-3: T cell immunoglobulin and mucin domain-containing protein 3
LAG-3: Lymphocyte-activation gene 3
FDA: The Food and Drug Administration
ER: Estrogen receptor
PR: Progesterone receptor
HER-2: Human epidermal growth factor receptor-2/neu
LABC: Locally advanced breast cancer
pCR: Pathologic complete response
MDACC: MD Anderson Cancer Center
SPSS: Statistical Package for Social Sciences
DFS: Disease-free survival
DSS: Disease-specific survival
